# Case of a Gun-Shot Wound in the Mouth; in Which, on Account of Impeded Deglutition, a Flexible Catheter Was Introduced through the Nose into the Oesophagus, and Suffered to Remain There during the Space of a Month

**Published:** 1791

**Authors:** 

**Affiliations:** Surgeon of the Hotel Dieu at Paris.


XVIII. *Cafe of a Gun-ſhot Wound in the Mouth; in which, on account of impeded Deglutition, a flexible Catheter was introduced through the Noſe into the Oeſophagus, and ſuffered to remain there during the Space of a Month. By M.* Manoury, *Surgeon of the Hotel Dieu at Paris.* Vide *Journal de Chirurgie, par M.* Default, *Chirurgien en Chef de L'Hotel Dieu de Paris.* Tome I. 8vo. Paris, 1791.

THIS very curious caſe, though deſcribed by M. Manoury, appears to have been chiefly under the direction of M. Default. It relates to a young man, who on the 18th of December, 1789, about midnight, diſcharged a loaded piſtol into his mouth. M. Default, who ſaw him within an hour after the accident, found him bleeding profuſely at the mouth, with his face already conſiderably ſwelled; the inſide of his mouth blackened by the powder; the right half of his tongue much torn and burnt; his lower jaw fractured on the right ſide; and a loſs of ſubſtance in the back part of the bony palate, on the ſame ſide, large enough to admit his thumb; together with a laceration of the velum pendulum palati.

In

In order to aſcertain the extent of the wound M. Default, we are told, introduced a female catheter through the opening in the palate. From this examination there did not ſeem to be any communication with the cavity of the ſkull; and that the brain was not injured appeared ſtill more clearly from the rational ſtate of the patient; but M. Default was unable to diſcover either of the three balls, with which the patient by ſigns made him to underſtand the piſtol had been loaded. They were not to be found in the blood which had been diſcharged, and the patient was certain that he had not ſwallowed them; it was therefore thought likely that they might be concealed in the cells of the os ethmoides or in the ſphenoidal ſinuſes. With a view to ſuppreſs the hæmorrhage the flexible ſilver wire of a catheter was introduced through the right noſtril into the fauces, and its extremity, by the aſſiſtance of a finger, was brought out at the mouth; which was an operation of ſome difficulty on account of the ſwelling of the parts. To this extremity were tied the ends of two pieces, or ribands, as the author calls them, of waxed thread, between which was faſtened a doſſil of lint, large enough to fill that part of the pharynx which correſponds with the poſterior noſtrils. By withdrawing the wire and threads through the noſe, the doſſil was carried into the fauces, and, by the aſſiſtance of a finger, applied againſt the poſterior opening of the noſtrils. The two portions of thread which came out through the noſe being then ſeparated, one of them was pulled towards the ſeptum narium and the other to the oppoſite ſide. The noſtril was now filled with ſmall doſſils of lint, over the laſt of which, larger than the reſt, were tied the two ends of the waxed threads.

After having thus put a ſtop to the hæmorrhage, M. Deſault attempted to reduce the two fragments of the lower jaw, one of which had been forced more than half an inch above the other; but the ſwelling of the ſoft parts rendered this attempt fruitleſs. He therefore, we are told, contented himſelf with applying to the cheeks, chin, and upper part of the neck, compreſſes moiſtened with vegeto-mineral water. But notwithſtanding the frequent renewal of this appplication, and the uſe of a ſuitable gargle, the tumefaction went on increaſing, ſo that the next day deglutition was become extremely painful and difficult, and on the ſecond was altogether impoſſible.

In this alarming ſtate of the caſe, M. Deſault was induced to remove the dossils of lint from the nostrils and fauces, as they were no longer necessary, and to introduce through the left nostril a large catheter made of elastic gum, and properly curved, which he had before employed with success, in a similar manner. Having carried this as far as the middle and posterior part of the pharynx, he with one hand drew out the wire of the catheter, while with the other he supported and fixed the catheter itself, which he endeavoured to introduce into the œsophagus, instead of which it passed, it seems, at first into the larynx. This was immediately known by a kind of guggling noise, and by the agitation of the flame of a candle brought close to the mouth of the catheter. Such a deviation, the author observes, in an attempt to introduce a flexible catheter in this manner into the œsophagus is frequent, as the surgeon seldom succeeds at once in getting it into that channel. The inconvenience, however, arising from such a deviation, is, he adds, not great; it being easy to discover it, not by the acute pain and convulsive cough, as hath been supposed (for in general neither of these, he remarks, takes place, and the patients appear to be but little incommoded by it) but by the trial with the flame of a candle, in the way juſt now de-ſcribed.

M. Default having inſtantly withdrawn the catheter from the larynx, made a freſh attempt to get it into the œſophagus and ſucceeded. It was ſecured by means of two waxed threads fixed to its outer extremity, and twiſted round a pin in each ſide of the patient's night cap. About four ounces of broth were now injected through the catheter into the ſtomach, and an attendant was inſtructed in the manner of repeating this operation occaſionally. In this way, it ſeems, ſuitable medicines and nouriſhment were introduced into the ſtomach with great facility and without exciting the leaſt ſickneſs or uneaſineſs. The patient, we are told, was apprized of the neceſſity of repeating them, not by the uſual ſymptoms of hunger and thirſt, but by a peculiar ſenſation of weakneſs and gnawing in the epigaſtric region which ceaſed as ſoon as the injection was repeated.

On the third day there was a conſiderable degree of fever, and the inſide of the mouth was filled with ſmall portions of ſloughs, which, on the fourth day, when a ſuppuration began to take place, were more eaſily detached and brought away by means of a gargle of barley water and honey of roſes, of which the patient was directed to make frequent uſe. Hitherto the ſwelling of the parts had gone on increaſing, and was now, we are told, ſo conſiderable that the fauces appeared, as it were, entirely cloſed. It would therefore have been impoſſible, our author obſerves, to have got down the leaſt ſubſtance either liquid or ſolid in the uſual way of ſwallowing, and without the aſſiſtance of the catheter, which continued to remain in the œſophagus without any inconvenience to the patient.

On the ſeventh day the ſwelling appeared to be a little diminiſhed; the fever alſo was leſſened; and the ſuppuration on the inſide of the mouth was conſiderable, and furniſhed a copious diſcharge of a grayiſh and fœtid pus, which rendered a frequent uſe of the gargle neceſſary.

On the fifteenth, the tumefaction of the cheeks and mouth being almoſt entirely diſſipated, M. Default made a freſh but unſucceſsful attempt to reduce the fracture of the lower jaw. The catheter was ſtill kept in the œſophagus, and the patient ſeemed to mend daily.

From the fifteenth to the twentieth day, nothing remarkable, we are told, occurred. The mouth, at this period, was pretty free from ſloughs, and and feveral parts of it which had been in a ftate of ulceration were already healed, as was alfo the velum pendulum palati; but there was ftill a hole in the roof of the mouth. The catheter appearing to be no longer neceffary, was now, it feems, withdrawn, and the patient attempted to fwallow a little broth; but the lofs of one half of his tongue, the cicatrices on the infide of his mouth and the conftriction they occafioned, together with the opening in the bony palate and his having been fo long unaccuftomed to fwallowing, all concurred in rendering deglutition fo difficult, that he entreated to have the ufe of the catheter continued fome days longer. It was accordingly again introduced and fuffered to remain till the thirtieth day, when it was finally withdrawn.

At firft, and for feveral days after its removal, deglutition was performed, it feems, with difficulty; but by degrees became more eafy. The patient's pronunciation likewife, we are told, was for fome time difficult and indiftinct.

The two fragments of the lower jaw were not yet united. One of them was ftill, it feems, confiderably higher than the other, though not fo much fo as at firft; and their reduction was again attempted, but with as little fuccefs as before.

The

The patient remained at Paris a month after the removal of the catheter, and at the end of that time went into the country to his relations. The fractured portions of the lower jaw were even then not consolidated, but were reduced nearly to a level with each other, and it was thought likely that by removing one of the dentes molares, which by its projection seemed now to be the chief obstacle to the complete reduction of the fracture, every difficulty with regard to it would gradually give way.

Instead of a hole in the bony palate, there was now, we are told, only a small fissure to be perceived, which there was reason to expect would soon be completely closed. The patient had recovered, in some measure, the sense of taste, and although he still masticated with difficulty, was able to take solid food, and could even chew a crust of bread. He articulated, however, with difficulty, and spoke through his nose, except when he wore spectacles so as to compress his nostrils to a certain degree.

In some reflections on this case M. Manoury points out the great advantages the patient derived from the use of the flexible catheter, which by conveying suitable food and medicines into the stomach, seemed to have been the chief means of preferving his life. The utility of fuch an inftrument fo applied, he obferves, is not confined to cafes fimilar to the prefent, but may be extended to a variety of other difeafes, fuch as tetanus, hydrophobia, fpafmodic contraction of the pharynx, paralyfis of its mufcles or of thofe of the tongue, and tumours fituated along the œfophagus or in its coats, even within the thorax. Nor are the advantages of thefe catheters, he contends, limited to difeafes that prevent deglutition, as they are capable of being employed with fuccefs in thofe which affect the channels of refpiration, whenever the obftacle is feated above the bronchia, as, for example, in cafes of abfcefs or ulceration of the inner furface of the larynx, with difeafe of the cartilages; in certain fiftulas of the trachea or larynx; in wounds of thofe parts, &c. He even goes fo far as to query, whether in cafes where both refpiration and deglutition are impeded at the fame time, as in fome fpecies of angina, in wounds of the neck where both the larynx and œfophagus are divided, it may not be advifable to introduce a flexible catheter through each noftril, and to pafs one into the œfophagus, and the other into the larynx, fixing them to the patient's cap, as in the preceding cafe, and taking care to diftinguifh them by fome obvious mark, fo that the injection may not by miftake be forced into the lungs inftead of the ftomach. M. Default, he obferves, has as yet made no trial of this method in affections of the larynx, but propofes to have recourfe to it in the firft favourable cafe that fhall prefent itfelf; and he forefees, we are told, nothing that can prevent it from fucceeding.

The facility with which thefe catheters may be introduced into the larynx, the little inconvenience fome perfons have experienced who have had them in that paffage for feveral minutes, and the effects of canulas which have been worn by patients in the trachea, feveral days after the operation of bronchotomy, all tend, he thinks, to obviate the objections which may be made to fuch a mode of treatment on account of the difficulty of executing it, or the fuppofed impoffibility of a patient's fupporting fuch an inftrument in a part thought to be fo irritable as the larynx.

